# Real-time associations among MS symptoms and cognitive dysfunction using ecological momentary assessment

**DOI:** 10.3389/fmed.2022.1049686

**Published:** 2023-01-12

**Authors:** Michelle H. Chen, Christine Cherian, Karen Elenjickal, Caroline M. Rafizadeh, Mindy K. Ross, Alex Leow, John DeLuca

**Affiliations:** ^1^Institute for Health, Health Care Policy and Aging Research, Rutgers University, New Brunswick, NJ, United States; ^2^Department of Neurology, Robert Wood Johnson Medical School, Rutgers University, New Brunswick, NJ, United States; ^3^Kessler Foundation, East Hanover, NJ, United States; ^4^Department of Physical Medicine and Rehabilitation, New Jersey Medical School, Rutgers University, Newark, NJ, United States; ^5^Department of Psychiatry, University of Illinois at Chicago, Chicago, IL, United States

**Keywords:** multiple sclerosis (MS), experience sampling, cognitive impairment, depression, anxiety, fatigue, pain, sleep

## Abstract

**Introduction:**

Multiple sclerosis (MS) is characterized by a wide range of disabling symptoms, including cognitive dysfunction, fatigue, depression, anxiety, pain, and sleep difficulties. The current study aimed to examine real-time associations between non-cognitive and cognitive symptoms (latter measured both objectively and subjectively in real-time) using smartphone-administered ecological momentary assessment (EMA).

**Methods:**

Forty-five persons with MS completed EMA four times per day for 3 weeks. For each EMA, participants completed mobile versions of the Trail-Making Test part B (mTMT-B) and a finger tapping task, as well as surveys about symptom severity. Multilevel models were conducted to account for within-person and within-day clustering.

**Results:**

A total of 3,174 EMA sessions were collected; compliance rate was 84%. There was significant intra-day variability in mTMT-B performance (*p* < 0.001) and levels of self-reported fatigue (*p* < 0.001). When participants reported depressive symptoms that were worse than their usual levels, they also performed worse on the mTMT-B (*p* < 0.001), independent of upper extremity motor functioning. Other self-reported non-cognitive symptoms were not associated with real-time performance on the mTMT-B [*p* > 0.009 (Bonferroni-corrected)]. In contrast, when self-reported fatigue (*p* < 0.001), depression (*p* < 0.001), anxiety (*p* < 0.001), and pain (*p* < 0.001) were worse than the individual’s typical levels, they also reported more severe cognitive dysfunction at the same time. Further, there was a statistical trend that self-reported cognitive dysfunction (not mTMT-B performance) predicted one’s self-reported sense of accomplishment in real-time.

**Discussion:**

The current study was the first to identify divergent factors that influence subjectively and objectively measured cognitive functioning *in real time* among persons with MS. Notably, it is when symptom severity was worse than the individual’s usual levels (and not absolute levels) that led to cognitive fluctuations, which supports the use of EMA in MS symptom monitoring.

## 1. Introduction

Multiple Sclerosis (MS) is a demyelinating, neurodegenerative disorder of autoimmune causes that disrupts the central nervous system (CNS). It is among the most common neurological diseases, and its age of onset typically occurs between 20 and 50 years (1). MS is accompanied by a range of symptoms, including cognitive dysfunction, fatigue, pain, mood changes, sleep problems, weakness, motor problems, and visual impairment (2).

Cognitive dysfunction, perhaps the most disabling manifestation of MS, is present in approximately 45–60% of MS cases (3, 4). Deficits in learning and memory as well as information processing speed are the most prevalent cognitive deficits in MS (5). Difficulties are also evidenced in complex attention, executive functioning, working memory, and visuospatial functions (5). Such impairments can affect everyday tasks of individuals with MS, disrupting their quality of life, overall wellbeing, and physical and social functioning (6).

Multiple sclerosis symptom severity can fluctuate throughout the day and week (7, 8), which is not captured by traditional clinical tools that ask patients to rate their average symptoms over a period of time (e.g., over the past week or month). The retrospective nature of these inventories can introduce recall bias (9), which is especially problematic for a population with memory difficulties such as MS. There is a need for real-time assessment of MS symptoms, which will improve our understanding of day-to-day symptom variability and inter-symptom associations, as well as advance the development of individualized MS treatment recommendations.

Ecological momentary assessment (EMA) is an approach that repeatedly samples an individual’s experiences in real time (e.g., asking them to report their symptom severity weekly, daily, or even every few hours) (10). By assessing real-time MS symptom severity several times per day, EMA allows for direct examination of within-person dynamics and diurnal symptom patterns. EMA has been widely used in studying behavioral health and psychological symptoms such as mood, addiction, and wellbeing (10). However, few MS studies have used this paradigm. Available, albeit limited, MS studies using EMA have shown good feasibility with relatively high compliance rates among their participants, ranging from 83 to 91% (7, 11). The current study will add to this emerging literature.

As with other MS symptoms, cognitive functioning is variable and can fluctuate on a daily basis due internal (e.g., stress) (12) or external triggers (e.g., temperature) (13). With the advent of mobile technology, cognitive assessment can now be easily administered through an individual’s smartphone. When combined with EMA, mobile cognitive testing permits the study of real-time associations among cognition, everyday tasks and environment, and other related symptoms (14). For example, an EMA study conducted in middle-aged and older adults with HIV found that engagement in cognitively stimulating activities was associated with better executive functioning and verbal learning, while engagement in more passive activities resulted in worse executive functioning and verbal learning performance (14).

Among the limited literature using EMA in MS, most investigations focused on fatigue. These studies have shown substantial within-person variability in fatigue intensity (7, 15), which justifies the use of EMA in this population. Only one research group has examined a broad range of symptoms, including cognitive dysfunction, depressed mood, fatigue, and pain, as well as inter-symptom associations (6, 7, 16). A study conducted by this group found that poorer cognitive functioning was preceded by worsening within-day pain and fatigue (16). However, cognitive functioning in this study was based on self-report. Given that studies have shown that self-reported cognitive dysfunction do not always correlate with objectively measured cognition (17, 18), more research is needed to clarify the associations between non-cognitive MS symptoms and both subjective and objective cognitive outcomes. Notably, the study found that it was only within-person changes (or “state”) in symptom ratings that were associated with other symptoms, and there were no cross-symptom associations in mean symptom levels across time points (or “trait”) (16). The state aspect of a symptom refers to transient fluctuations at a point in time that can be affected by situational contexts (e.g., being more anxious than usual because of a doctor’s appointment). The trait aspect of a symptom represents the typical pattern for an individual (e.g., usual levels of anxiety) (19). Given the high sampling frequency, EMA enables such separations.

The current study aimed to use smartphone-administered EMA to investigate and characterize real-time relationships between non-cognitive and cognitive symptoms among persons with MS. We expect that deviations in non-cognitive MS symptoms from individuals’ typical levels will be associated with real-time cognitive changes. The current study will address the limitations of prior studies by measuring cognitive functioning both objectively and subjectively.

## 2. Materials and methods

### 2.1. Participants

Participants were recruited through online advertisements on the National MS Society and Kessler Foundation websites and social media. Interested prospective participants would contact the research team and undergo a brief phone screening to ascertain eligibility. Inclusion criteria included: (1) ownership of an iPhone, (2) access to a desktop or laptop computer that is at least 13 inches in screen size, (3) English is primary language, and (4) self-reported diagnosis of MS by a medical professional. Exclusion criteria consisted of: (1) self-reported diagnosis of neurological conditions other than MS, (2) self-reported diagnosis of serious mental illness (e.g., schizophrenia, bipolar disorder), (3) self-reported diagnosis of attention-deficit/hyperactive disorder or specific learning disorder, (4) self-reported problems with substance misuse, (5) presence of sensory or motor difficulties that would interfere with validity of study assessments (self-reported and through examiner observation), and (6) self-reported MS relapse/exacerbation symptoms within the month prior to enrollment. The study was approved by the Kessler Foundation Institutional Review Board. All participants provided electronic written informed consent through Research Electronic Data Capture (REDCap) tools (20, 21), hosted by New Jersey Medical School, Rutgers University.

### 2.2. Procedures

Data collection took place between April 2021 and February 2022. Due to the coronavirus disease 2019 (COVID-19) pandemic (22), all study procedures were conducted virtually. Participants completed a virtually administered neuropsychological battery and self-report inventories at baseline. After the baseline assessment, they were instructed on downloading and using the study app (23–25). Then participants were asked to complete EMAs four times per day for 3 weeks. EMAs consisted of brief self-report ratings and performance-based tasks delivered through the participant’s smartphone.

### 2.3. Baseline assessment

Participants completed a virtually administered baseline assessment (*via* videoconferencing), which consisted of a brief battery of neuropsychological tests and phone-based Expanded Disability Status Scale (EDSS) (26). The neuropsychological battery included the oral version of the Symbol Digit Modalities Test (SDMT) (27), which is considered a gold standard clinical trial endpoint for MS-related cognitive dysfunction (28) and was used in the current study to characterize cognitive status (other neuropsychological measures were not used and therefore omitted in this paper). On the SDMT, participants were provided with a key of nine symbol-digit pairs. They were instructed to call out numbers associated with symbols presented in the test stimulus set one at a time as quickly as they could within 90 s. SDMT measures processing speed, with higher scores indicating faster processing speed. Raw scores for SDMT were converted to *z*-scores using normative data from Strober et al. (29). For the phone version of EDSS, assessment of ambulation and functional systems were obtained *via* self-report based on procedures outlined in Lechner-Scott et al. (26). EDSS is the standard method for assessing neurological disability among persons with MS and ranges between 0 and 10 with 0.5 increments (e.g., 0 = no disability, 2.5 = mild disability, 6.0 = requiring a walking aid, 9.0 = confined to bed).

### 2.4. Ecological momentary assessment protocol

**FIGURE 1:**
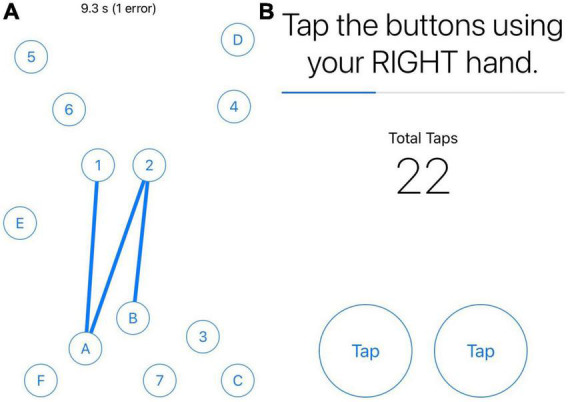
Screenshots of mTMT-B and finger tapping tasks. Panel **(A)** is screenshot of the mobile Trail-Making Test part B (mTMT-B) task. Panel **(B)** is a screenshot of the mobile finger tapping task. Users performed one trial with their right hand, followed by a second with their left hand.

EMAs were administered using the BiAffect app (23–25), which was available for download for iOS devices through the Apple app store. There were four EMAs per day during the 3-week monitoring period. The first three EMAs each day focused on self-reported symptom severity in real-time and performance on smartphone-based cognitive and motor tasks (see Table 1 for details on EMA measures used in this study) including part B of the Trail-Making Test (mTMT-B) (25) and a finger tapping task (30). For the mTMT-B task, participants were asked to connect and alternate between numbers and letters consecutively and quickly on the screen (see Figure 1A). For the mobile finger tapping task, participants were asked to tap two fingers of the same hand alternatively as quickly as possible for 10 s; they performed one trial with their right hand and another trial with their left hand (see Figure 1B). Symptom severity was based on the Visual Analogue Scale (VAS) (31), which is commonly used in EMA research, including EMA studies conducted in MS (6, 7, 11). The last EMA of the day asked for reports that only required one response per day (e.g., sleep, sense of accomplishment). Throughout the monitoring period, participants were prompted to complete EMAs through text messages (with reminders to complete them on the study app). They were told to complete each EMA within 1 h of the prompt if not exactly at the prompted time. The first three EMAs were approximately equally spaced in time throughout the day (first in the morning, second in mid-day/early afternoon, and third in late afternoon/early evening) based on the participant’s individual sleep-wake cycle. If a participant had a different schedule for the weekend, their EMA schedule was adjusted accordingly. The last EMA of the day was administered about 1–2 h before the participant’s bedtime.

**TABLE 1:** Ecological momentary assessment protocol.

Construct	Question/task	Assessment modality	Administration frequency
Fatigue	What is your level of fatigue right now on a scale of 0 (no fatigue)—10 (extremely severe fatigue)?	Self-report	3×/day
Depression	What is your level of depression right now on a scale of 0 (not at all depressed)—10 (extremely depressed)?	Self-report	3×/day
Anxiety	What is your level of anxiety right now on a scale of 0 (not at all anxious)—10 (extremely anxious)?	Self-report	3×/day
Upper extremity weakness	What is your level of upper extremity weakness on a scale of 0 (no weakness)—10 (extremely severe weakness)?	Self-report	3×/day
Pain	What is your level of pain right now on a scale of 0 (no pain)—10 (worst pain imaginable)?	Self-report	3×/day
Overall cognitive function	What is your level of cognitive function right now on a scale of 0 (good: my thinking is sharp and quick)—10 (bad: my thinking is very difficult or slow)?	Self-report	3×/day
Executive function	Mobile version of the Trail-Making Test part B (25); participants connected consecutive numbers and letters in order; completion time was used as primary outcome in multilevel models	Performance-based	3×/day
Upper extremity motor speed	Mobile version of a Finger Tapping task (30); participants tapped two fingers of the same hand alternatively for 10 s as quickly as possible; this was done for both right and left hands; average number of taps across two hands was used as covariates in multilevel models	Performance-based	3×/day
Sleep	How many hours of sleep did you get last night? Did you have difficulty falling asleep (yes or no)?	Self-report	1×/day
Accomplishment	To what extent were you able to accomplish everything you wanted to do today on a scale of 0 (I was unable to accomplish anything I wanted to do today)—10 (I was able to accomplish everything I wanted to do today)?	Self-report	1×/day

### 2.5. Statistical analysis

All analyses were conducted in R version 4.2.1. Descriptive statistics were used to determine demographic and clinical characteristics of the sample. Multilevel models were used to examine intraday variability and associations among EMA measures, in order to account for within-subject and within-day clustering. All multilevel models included random intercepts for the subject (to account for within-person clustering) as well as random intercepts for concatenation of subject and day variables (e.g., day 1 for subject 0001 is 00011, day 2 for subject 0001 is 00012, etc.; to account for within-day clustering) (32), except for variables collected for only once per day (i.e., sleep, sense of accomplishment) which only included the subject’s intercept. All models were fit using the restricted maximum likelihood approach, which is the recommended default method by the R packages lme4 (33) and lmerTest (34).

#### 2.5.1. Compliance to EMA and intraclass correlations

For the first three EMAs of each day (which were time-sensitive), we included the responses in the final dataset if the EMA was completed within 2 h before or after the scheduled time. If participants completed multiple EMA measures within each scheduled period, the first complete response was used. For the last EMA of the day (not time-sensitive), we used the first complete response submitted after the third time-sensitive EMA. Compliance was defined as the ratio of completed EMAs within the specified time periods out of the total number of required EMAs. ICCs for each symptom rating and performance was calculated based on the null (unconditional) multilevel models (with only the random intercepts without fixed effects). ICCs signified the proportions of between-person (in this case, random variance for the subject ID variable) and between-day (in this case, random variance for the concatenated subject and day variable) variances out of the total random variance for each outcome.

#### 2.5.2. Separating state and trait aspects of symptom rating/performance

We separated state (how each symptom varied from the individual’s typical level) and trait (each individual’s typical level of symptom severity) aspects of each symptom rating/performance score using participant-mean centering (35). First, for each symptom rating/performance, the scores for different EMA sessions were averaged within each participant, creating the participant means which were also the trait aspect of that symptom rating/performance (e.g., each individual’s typical level of depressive symptoms). Then, we centered each EMA score around the participant mean; this was the state aspect of each symptom rating/performance for each EMA session (e.g., when depressive symptoms were more or less severe than the individual’s typical level of depression). For self-reported difficulties falling asleep, since it is a binary variable (not continuous), we did not separate their state and trait aspects because there were no “participant means.”

#### 2.5.3. Intra-day fluctuations in symptom severity

Multilevel models were conducted to evaluate symptom fluctuations over time. In these models, each MS symptom rating as well as mTMT-B completion time were outcomes, and session number was fixed effect predictor. For mTMT-B, the model was adjusted for age, mean bilateral finger tapping performance (number of taps), state and trait upper extremity weakness rating, and measurement number (to account for practice effects). For self-reported symptom ratings other than depressive symptoms (self-reported cognitive dysfunction, fatigue, anxiety, pain, and sleep), models were adjusted for state and trait depressive symptom ratings (to account for response bias due to depression).

#### 2.5.4. Real-time associations between symptom ratings and cognitive functioning in real time

Multilevel models were used to determine real-time associations between non-cognitive symptom ratings (fatigue, depression, anxiety, pain, and sleep; state and trait aspects of each symptom as well as their interactions as fixed effect predictors in each model) and measures of cognition (mTMT-B completion time and self-reported cognitive dysfunction; each as outcome in separate model). As in previous models, models with mTMT-B completion time as outcome were adjusted for age, mean bilateral finger tapping performance, state and trait upper extremity weakness rating, and measurement number. Models with self-reported cognitive dysfunction rating as outcome were adjusted for state and trait depressive symptom ratings.

#### 2.5.5. Real-time associations between cognitive functioning and self-reported sense of accomplishment in real time

Multilevel models were used to examine real-time associations between cognitive functioning (mTMT-B and self-reported cognitive dysfunction) and perceived sense of accomplishment, with state and trait aspects of the former as fixed effect predictors and latter as outcome. Models were adjusted for state and trait depressive symptom ratings.

#### 2.5.6. Multiple comparison corrections

Since each set of analyses answered an independent question, we adjusted for multiple comparisons using Bonferroni correction for each outcome separately (instead of adjusting for all models conducted in the study). For intra-day variation in symptoms (section “2.5.3. Intra-day fluctuations in symptom severity”), associations between non-cognitive symptoms and mTMT-B performance (section “2.5.4. Real-time associations between symptom ratings and cognitive functioning in real time”), and associations between non-cognitive symptoms and self-reported cognitive dysfunction (section “2.5.4. Real-time associations between symptom ratings and cognitive functioning in real time”), six models were conducted for each question, so the Bonferroni-corrected *p*-value threshold is 0.05/6 = 0.009. For predictors of sense of accomplishment (section “2.5.5. Real-time associations between cognitive functioning and self-reported sense of accomplishment in real time”), two models were conducted, so the Bonferroni-corrected *p*-value threshold s 0.05/2 = 0.025.

## 3. Results

The study sample consisted of 45 participants with MS, who completed 3,174 EMA sessions across the 3-week monitoring period. Compliance to EMA was 84%. Table 2 summarizes demographic and clinical characteristics of the sample. The sample was, on average, middle-aged and consisted of primarily females and non-Hispanic whites. Majority of the sample completed at least some college. Relapsing-remitting disease course was the dominant phenotype. Disease duration was heterogenous, ranging between several months to almost 30 years. Based on self-report, most participants had EDSS scores between 2.5 and 4.5, which signified the ability to ambulate without aid with some degrees of limitation. Compared to a normative sample, participants in this study had mild to moderate processing speed impairment (*z*-score approaching 1.5 standard deviations below the mean). Between-person and between-day ICCs based on unconditional multilevel models for each symptom rating/performance are summarized in Table 3. Across symptoms, approximately two-thirds of the random variance was attributed to between-person variability relative to within-person variability. Between-day variability was small within each person.

**TABLE 2:** Demographic and clinical characteristics of the sample.

Age: mean years (SD); range	41.69 (13.39); 20–70
**Sex**	
Female	41 (91.11)
Male	4 (8.89)
**Education: number (proportion)**	
High school graduate or fewer years of education	4 (8.89)
Some college with no degree or associate’s degree	12 (26.67)
Bachelor’s degree	17 (37.78)
Master’s degree	10 (22.22)
Doctoral degree	1 (2.22)
Prefer not to answer	1 (2.22)
**Race/ethnicity: number (proportion)**	
Non-Hispanic white	33 (73.33)
Non-Hispanic black	5 (11.11)
Hispanic/Latino(a)	3 (6.67)
Asian	3 (6.67)
Prefer not to answer	1 (2.22)
**MS disease course: number (proportion)**	
Relapsing-remitting	39 (86.67)
Primary progressive	3 (6.67)
Secondary progressive	2 (4.44)
Not sure	1 (2.22)
MS disease duration: mean years (SD); range	11.06 (9.30); 4.38 months—29.95 years
**EDSS**	
0–2: number (proportion)	5 (11.11)
2.5: number (proportion)	12 (26.67)
3.0: number (proportion)	14 (31.11)
3.5–4.5: number (proportion)	11 (24.45)
> 4.5: number (proportion)	3 (6.66)
SDMT: *z*-score (SD)	-1.46 (1.34)

MS, multiple sclerosis; SD, standard deviation; EDSS, Expanded Disability Status Scale; SDMT, Symbol Digit Modalities Test.

**TABLE 3:** Intraclass correlations (ICCs) for each symptom rating/performance.

	Between-person ICC	Between-day ICC within persons
mTMT-B competition time	0.62	0.11
Self-reported cognitive dysfunction	0.59	0.17
Self-reported fatigue	0.62	0.07
Self-reported depressive symptoms	0.66	0.17
Self-reported anxiety	0.64	0.15
Self-reported pain	0.76	0.08
Self-reported number of hours slept	0.54	N/A
Self-reported difficulties falling asleep	0.64	N/A

ICCs were calculated based on null (unconditional) models with only subject and day random intercepts. Since sleep questions were only administered once per day, there were no between-day ICCs.

### 3.1. Intra-day fluctuations in symptom severity

**FIGURE 2:**
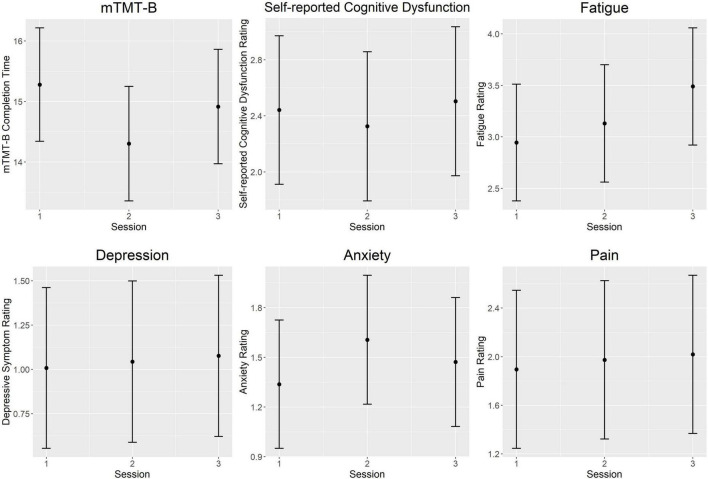
Intra-day fluctuations of symptom severity. Both objectively and subjectively measured cognition was worse in the morning and end of day compared to the middle of the day. Anxiety ratings showed the opposite trend and peaked at mid-day. Fatigue ratings increased steadily throughout the day. There were no significant intra-day variations in ratings of pain and depressive symptoms. Each plot represents predicted values from multilevel models for the session fixed effect. Error bars represent 95% confidence intervals. mTMT-B, mobile Trail-Making Test part B.

Figure 2 illustrates intra-day fluctuations in various MS symptom severity, and Table 4 summarizes the associated model estimates. mTMT-B completion time and fatigue ratings showed the most variation across sessions each day. Anxiety ratings showed significant variation in the earlier part of the day (sessions 1 vs. 2), while self-reported cognitive dysfunction showed significant variation in the latter part of the day (sessions 2 vs. 3). Depression and pain ratings did not significantly vary across the day.

**TABLE 4:** Model estimates for intra-day symptom fluctuations.

Contrast	Standardized coefficient	95% confidence intervals	*P*-value
**mTMT-B completion time**			
Session 2 vs. session 1	-0.17	−0.23 to −0.11	< 0.001*
Session 3 vs. session 1	-0.06	−0.12 to 0.00	0.041
Session 3 vs. session 2	0.10	0.04 to 0.17	< 0.001*
**Self-reported cognitive dysfunction**			
Session 2 vs. session 1	-0.05	−0.10 to 0.01	0.076
Session 3 vs. session 1	0.03	−0.03 to 0.08	0.334
Session 3 vs. session 2	0.07	0.02 to 0.13	0.008*
**Self-reported fatigue**			
Session 2 vs. session 1	0.07	0.01 to 0.13	0.025
Session 3 vs. session 1	0.21	0.15 to 0.27	< 0.001*
Session 3 vs. session 2	0.14	0.07 to 0.20	< 0.001*
**Self-reported depressive symptoms**			
Session 2 vs. session 1	0.02	−0.03 to 0.08	0.412
Session 3 vs. session 1	0.04	−0.01 to 0.10	0.111
Session 3 vs. session 2	0.02	−0.03 to 0.08	0.461
**Self-reported anxiety**			
Session 2 vs. session 1	0.12	0.07 to 0.18	< 0.001*
Session 3 vs. session 1	0.06	0.01 to 0.11	0.020
Session 3 vs. session 2	-0.06	−0.12 to -0.01	0.025
**Self-reported pain**			
Session 2 vs. session 1	0.03	−0.01 to 0.07	0.162
Session 3 vs. session 1	0.05	0.01 to 0.09	0.026
Session 3 vs. session 2	0.02	−0.03 to 0.06	0.436

All models included random intercepts for subject and an aggregated subject and day variable. Models with mTMT-B as outcomes included age, mean bilateral finger tapping performance, state and trait upper extremity weakness rating, and measurement number as fixed effects. Models with self-reported symptom ratings other than depressive symptoms included state and trait depressive symptom ratings as fixed effects. mTMT-B, mobile Trail-Making Test part B. *Denotes significant comparisons at Bonferroni-corrected *p* = 0.008 level.

### 3.2. Real-time associations between symptom ratings and cognitive functioning

**FIGURE 3:**
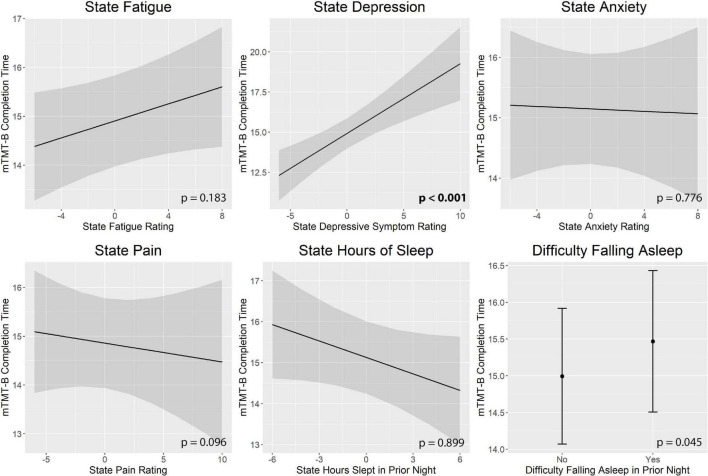
Real-time associations between non-cognitive symptom ratings and mTMT-B performance. Among all non-cognitive symptom ratings, only more severe state depressive symptoms was associated with slower mTMT-B completion time. State and trait aspects of each symptom was tested together in the same model; plots show the marginal effects of the state variables. All models included subject and concatenated subject and day variable as random intercepts; and age, mean bilateral finger tapping performance, state and trait upper extremity weakness rating, and measurement number as fixed effects. mTMT-B, mobile Trail-Making Test part B. Error bands represent 95% confidence intervals.

**FIGURE 4:**
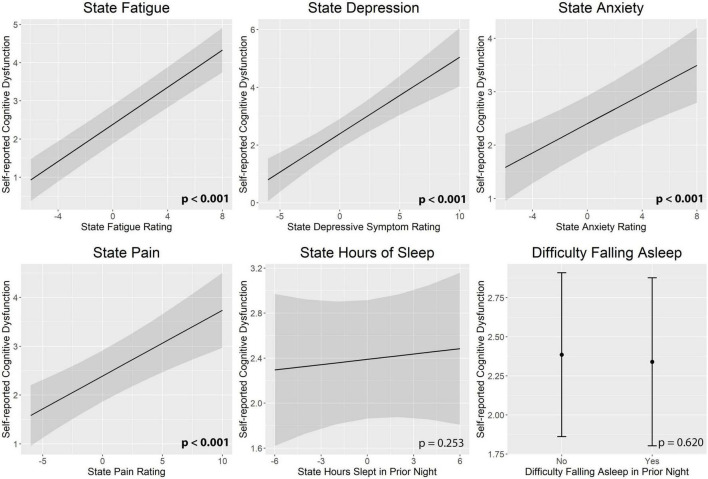
Real-time associations between non-cognitive symptom ratings and self-reported cognitive dysfunction. State fatigue, depressive symptoms, anxiety, and pain were all significant predictors of self-reported cognitive dysfunction. State and trait aspects of each symptom was tested together in the same model; plots show the marginal effects of the state variables. All models included subject and day number as random intercepts and state and trait depressive symptoms as fixed effects. Error bands represent 95% confidence intervals.

Figure 3 illustrates real-time associations between non-cognitive symptom ratings and mTMT-B performance, and Table 5 summarizes partial model estimates for the state and trait symptom variables (see Supplementary Table 1 for full model estimates). Figure 4 illustrates real-time associations between non-cognitive symptom ratings and self-reported cognitive dysfunction rating, and Table 6 summarizes partial model estimates for the state and trait symptom variables (see Supplementary Table 2 for full model estimates). Among all non-cognitive symptom ratings (both state and trait), only more severe state depressive symptoms were associated with slower mTMT-B completion time. On the other hand, for self-reported cognitive dysfunction, state fatigue, depressive symptoms, anxiety, and pain were all significant predictors, with higher severity in non-cognitive symptoms correlating with more severe self-reported cognitive dysfunction. None of the trait symptom levels, except for depressive symptoms, were significantly associated with self-reported cognitive dysfunction.

**TABLE 5:** Model estimates for real-time associations between non-cognitive symptom ratings and mTMT-B performance.

Variable	Standardized coefficient	95% confidence intervals	*P*-value
State fatigue	0.03	−0.004 to 0.05	0.183
Trait fatigue	−0.05	−0.21 to 0.12	0.581
State depressive symptoms	0.08	0.04 to 0.12	< 0.001*
Trait depressive symptoms	−0.04	−0.19 to 0.11	0.605
State anxiety	−2.44 × 10^–03^	−0.04 to 0.03	0.776
Trait anxiety	−0.13	−0.30 to 0.05	0.160
State pain	−8.14 × 10^–03^	−0.04 to 0.02	0.096
Trait pain	−0.09	−0.29 to 0.10	0.349
State number of hours slept in prior night	−0.02	−0.05 to 0.00	0.899
Trait number of hours slept in prior night	−0.12	−0.26 to 0.02	0.104
Difficulties falling asleep in prior night (yes vs. no) in prior night	0.08	0.002 to 0.16	0.045

State and trait aspects of each symptom was tested together in the same model (along with their interaction). All models included random intercepts for subject and an aggregated subject and day variable; and age, mean bilateral finger tapping performance, state and trait upper extremity weakness rating, and measurement number as fixed effects. mTMT-B, mobile Trail-Making Test part B. *Denotes significant comparisons at Bonferroni-corrected *p* = 0.008 level.

**TABLE 6:** Model estimates for real-time associations between non-cognitive symptom ratings and self-reported cognitive dysfunction.

Variable	Standardized coefficient	95% confidence intervals	*P*-value
State fatigue	0.17	0.15 to 0.20	< 0.001*
Trait fatigue	0.20	−0.03 to 0.42	0.066
State depressive symptoms	0.12	0.08 to 0.16	< 0.001*
Trait depressive symptoms	0.27	0.10 to 0.43	0.005*
State anxiety	0.08	0.05 to 0.11	< 0.001*
Trait anxiety	−0.08	−0.37 to 0.20	0.493
State pain	0.07	0.04 to 0.10	< 0.001*
Trait pain	−0.03	−0.27 to 0.21	0.806
State number of hours slept in prior night	6.61 × 10^–03^	−0.02 to 0.04	0.253
Trait number of hours slept in prior night	−0.07	−0.27 to 0.13	0.361
Difficulties falling asleep in prior night (yes vs. no) in prior night	−0.02	−0.10 to 0.06	0.620

State and trait aspects of each symptom was tested together in the same model (along with their interaction). All models included random intercepts for subject and an aggregated subject and day variable (except for sleep variables which only included the subject intercept) and state and trait depressive symptoms as fixed effects. *Denotes significant comparisons at Bonferroni-corrected *p* = 0.008 level.

### 3.3. Real-time associations between cognitive functioning and perceived sense of accomplishment in real time

There was a statistical trend of lower level of state self-reported cognitive dysfunction (but not trait) correlating with higher perceived sense of accomplishment. Neither state nor trait mTMT-B completion time was associated with perceived sense of accomplishment. See Table 7 for model estimates.

**TABLE 7:** Model estimates for real-time associations between cognitive functioning and self-reported sense of accomplishment.

Variable	Standardized coefficient	95% confidence intervals	*P*-value
State mTMT-B completion time	−3.17 × 10^–03^	−0.04 to 0.04	0.608
Trait mTMT-B completion time	−0.15	−0.34 to 0.04	0.120
State self-reported cognitive dysfunction	0.04	0.00 to 0.07	0.027*
Trait self-reported cognitive dysfunction	−0.14	−0.35 to 0.07	0.205

State and trait cognitive functioning was tested together in the same model. All models included subject as random intercepts and state and trait depressive symptoms as fixed effects. *Denotes statistical trend (at Bonferroni-corrected *p* = 0.025 level).

## 4. Discussion

The current study examined real-time cognitive functioning among persons with MS using EMA. It is the first MS EMA study to include objectively measured cognitive functioning—in this case, executive functioning—in real-time. We found that when fatigue, depression, anxiety, and pain were more severe than the individual’s usual levels (“state” as opposed to “trait”), the individual reported more cognitive dysfunction. In contrast, objectively measured executive functioning seemed specifically sensitive to state depressive symptoms. Further, there was a trend that self-reported cognitive dysfunction predicted lower perceived sense of accomplishment more than objectively measured executive dysfunction. These results demonstrated divergent factors that influence subjectively and objectively measured cognitive functioning in real time and is the first of such investigation in the MS population. Our results confirmed cross-sectional studies linking cognition with fatigue, depression, anxiety, and pain among persons with MS (36–40), and further extended these studies by establishing real-time associations (more temporally precise) within the real-life context (more ecologically valid).

Results of the current study illustrated the importance of assessing state, and not just trait, aspect of each symptom when considering inter-symptom relationships. We found many significant associations with state variables and almost none with trait variables. This may explain why there were inconsistent findings among cross-sectional studies (focusing on trait), where sometimes certain MS symptoms were associated with other symptoms and sometimes such associations were absent. Only an EMA framework enables investigations into state variations in symptoms. This study confirmed the feasibility of utilizing EMA to assess a range of MS symptoms within the real-world context and showed comparable compliance rates (> 80%) as previous, albeit limited number of, studies (7, 11). Thus, it may be feasible to integrate this form of assessment into routine clinical practice. Current standard of MS care involves once-per-year evaluations, which do not take into account of symptom variability between visits. Even when providers ask about these variations, the responses are likely influenced by recall bias, especially for a population with known memory impairment such as MS. Other disciplines such as sleep medicine have already demonstrated the clinical feasibility and utility of EMA in the form of sleep diaries that patients have to complete daily for 1–2 weeks. Therefore, it is feasible for such practice to be integrated into MS care, particularly with aid from mobile technologies.

Remote monitoring of neurologic and cognitive symptoms using EMA and smartphone-based cognitive assessment may be extended to other populations as well. Such investigations have already begun in populations such as individuals with HIV (41) and Parkinson’s disease (42). Besides subjective EMA surveys and smartphone-based cognitive assessments, objective data on motor fluctuations (43) and psychological symptoms (44) can also be gathered using smartphone sensors in the ambulatory setting. These methods are not dissimilar to established remote monitoring practices used in cardiac (e.g., Holtzer monitor) and diabetes (e.g., continuous glucose monitoring) care. In the age of personalized medicine, remote monitoring will provide patients and clinicians with real-world, temporally rich data needed for individualized treatments and recommendations.

Inclusion of both subjectively and objectively measured cognitive functioning is a strength of the current study. Previous MS studies have found that subjective and objective cognitive functioning do not always correlate (17, 18), and subjective appraisal of one’s own cognition relates more strongly to affective symptomology (especially depression) than objective performance (18, 45). Our results help delineate differential factors that influence subjective and objective cognitive outcomes in real time. Further, given the known association between depression and subjective symptom reports (18, 45), we controlled for depression (both state and trait) in analyses with subjective symptom reports as outcomes. Thus, we can conclude that in addition to the clear associations between state depressive symptoms and cognitive dysfunction, state fatigue, anxiety, and pain symptoms were also related to self-reported cognitive deterioration in real time, independent of depression status.

In contrast, objectively measured executive dysfunction was only related to state depressive symptoms, independent of upper extremity motor functioning. It is possible that the self-report question captured a broader sense of cognitive dysfunction than the objective measure (mTMT) which specifically tapped into processing speed and executive functioning. Our results were consistent with prior MS literature that found a particularly unique association between depression and executive functioning (46–48).

Only one research group has examined a range of MS symptoms using EMA with an adequate sample size (6, 7, 16), while others focused on fatigue (11, 15, 49) or had a very limited sample size (8). We found that fatigue most consistently increased in severity throughout the day, which was consistent with a prior study (7). Further, we found significant inter-symptom associations especially among self-report measures, which was also concordant with the prior literature (16). Compared to works by this research group (6, 7, 16), the present research further added an objective cognitive measure and self-report of anxiety symptoms, which had not been previously investigated using EMA in MS.

Of note, the current study focused on deviations in symptom severity from the individual’s typical level (state vs. trait) and not absolute levels. This is an important context for the objective cognitive outcome (mTMT-B) used in this study because we were unable to determine whether individual instances of decline were clinically significant. In fact, there are currently no well-validated mobile cognitive measures with robust norms that take into account of repeated measurements and allow users to determine level of clinical impairment or decline. This is an area requiring future investigations. One promising effort is the National Institute on Aging (NIA)-funded Mobile Toolbox (50), which consists of a suite of mobile tasks validated against gold standard measures with population norms generated. The project is currently in its beta testing phase and will be eventually made available to external researchers. That being said, the current study’s version of mTMT-B has been validated with the traditional paper-and-pencil version of TMT in a small sample (25), and practice effects were accounted for in our analyses. For subjective measures, we used the VAS (31) as frequently used in other EMA investigations. But unlike cross-sectional self-report measures with established clinical ranges, severity levels as determined by the VAS are individualized and their relations to other disease characteristics are unknown. Thus, we focused on changes in symptom severity from individual’s typical levels as determined by the VAS.

The current study is limited by the relatively low levels of symptom severity reported by our sample. On average, participants were reporting symptom levels below 4 on a 10-point scale. This may be due to the fact that many of our participants had relatively chronic and stable disease course. Future studies should aim to recruit participants with more active disease in order to fully capture intra-day clinical fluctuations. That being said, even with relatively low levels of symptom severity, we still found significant intra-day fluctuations in objective cognitive performance and fatigue ratings.

Anther limitation is the predominance of female sex and relapsing-remitting disease course within our sample, which is consistent with prevalence rates in the general MS population but may restrict our ability to generalize our findings to minority populations such as males with MS and those with progressive disease courses. Future studies may consider oversampling these minority groups to confirm our findings.

Further, there may be a selection bias in our sample since only iPhone users were eligible for our study. While smartphone use is fairly ubiquitous in the U.S. [85% of Americans own smartphones (51)], there may be socioeconomic differences among individuals who use iPhones compared to Android devices. Finally, while EMA is advantageous over retrospective self-report because it minimizes recall bias, it is important to note that besides the mTMT-B and finger tapping tasks, all other symptoms were evaluated subjectively. Future studies may explore real-time objective measures for mood and fatigue through smartphone (e.g., GPS, call/text logs) and other wearable sensors (e.g., heart rate, skin conductance, sleep patterns).

In conclusion, the current study was the first to identify divergent factors that influence subjectively and objectively measured cognitive functioning in real time. While self-reported cognitive dysfunction was associated with a range of non-cognitive symptoms and self-reported sense of accomplishment, objectively measured executive functioning was only associated with depressive symptoms. Notably, we found that only state aspects of non-cognitive MS symptoms (and not trait) were associated with cognitive fluctuations, which supports the use of EMA in MS symptom monitoring.

## Data availability statement

The raw data supporting the conclusions of this article will be made available by the authors, without undue reservation.

## Ethics statement

The studies involving human participants were reviewed and approved by the Kessler Foundation. The patients/participants provided their written informed consent to participate in this study.

## Author contributions

MC designed the study, obtained the funding, analyzed the data, and wrote the full draft of the manuscript (except for section “1. Introduction”). CC wrote the section “1. Introduction” of the manuscript. KE and CR performed the data cleaning and calculations. MR managed and processed the study app data. AL headed the team that created the study app and managed app data, obtained the funding, and provided the feedback on data analysis and manuscript draft. JD obtained the funding and provided the feedback for the analysis and manuscript draft. All authors contributed to the article and approved the submitted version.

## References

[1] Mirmosayyeb O Brand S Barzegar M Afshari-Safavi A Nehzat N Shaygannejad V Clinical characteristics and disability progression of early-and late-onset multiple sclerosis compared to adult-onset multiple sclerosis. *J Clin Med.* (2020) 9:1326. 10.3390/jcm9051326 32370288 PMC7290335

[2] Crayton H Rossman H. Managing the symptoms of multiple sclerosis: a multimodal approach. *Clin Ther.* (2006) 28:445–60. 10.1016/j.clinthera.2006.04.005 16750459

[3] Chiaravalloti N DeLuca J. Cognitive impairment in multiple sclerosis. *Lancet Neurol.* (2008) 7:1139–51. 10.1016/S1474-4422(08)70259-X 19007738

[4] Guimarães J Sá M. Cognitive dysfunction in multiple sclerosis. *Front Neurol.* (2012) 3:74. 10.3389/fneur.2012.00074 22654782 PMC3359427

[5] Grzegorski T Losy J. Cognitive impairment in multiple sclerosis–a review of current knowledge and recent research. *Rev Neurosci.* (2017) 28:845–60. 10.1515/revneuro-2017-0011 28787275

[6] Kratz A Braley T Foxen-Craft E Scott E Murphy IJ Murphy S. How do pain, fatigue, depressive, and cognitive symptoms relate to well-being and social and physical functioning in the daily lives of individuals with multiple sclerosis? *Arch Phys Med Rehabil.* (2017) 98:2160–6. 10.1016/j.apmr.2017.07.004 28729170 PMC5660943

[7] Kratz A Murphy S Braley T. Ecological momentary assessment of pain, fatigue, depressive, and cognitive symptoms reveals significant daily variability in multiple sclerosis. *Arch Phys Med Rehabil.* (2017) 98:2142–50. 10.1016/j.apmr.2017.07.002 28729168 PMC5660933

[8] Kasser S Goldstein A Wood P Sibold J. Symptom variability, affect and physical activity in ambulatory persons with multiple sclerosis: understanding patterns and time-bound relationships. *Disabil Health J.* (2017) 10:207–13. 10.1016/j.dhjo.2016.10.006 27814947

[9] Raphael K. Recall bias: a proposal for assessment and control. *Int J Epidemiol.* (1987) 16:167–70. 10.1093/ije/16.2.167 3610443

[10] Wrzus C Neubauer A. Ecological momentary assessment: a meta-analysis on designs, samples, and compliance across research fields. *Assessment.* (2022): [Epub ahead of print]. 10.1177/10731911211067538 35016567 PMC9999286

[11] Powell D Liossi C Schlotz W Moss-Morris R. Tracking daily fatigue fluctuations in multiple sclerosis: ecological momentary assessment provides unique insights. *J Behav Med.* (2017) 40:772–83. 10.1007/s10865-017-9840-4 28281106 PMC5613039

[12] McEwen B Sapolsky R. Stress and cognitive function. *Curr Opin Neurobiol.* (1995) 5:205–16. 10.1016/0959-4388(95)80028-X 7620309

[13] Leavitt V Sumowski J Chiaravalloti N DeLuca J. Warmer outdoor temperature is associated with worse cognitive status in multiple sclerosis. *Neurology.* (2012) 78:964–8. 10.1212/WNL.0b013e31824d5834 22402861 PMC3310313

[14] Campbell L Paolillo E Heaton A Tang B Depp C Granholm E Daily activities related to mobile cognitive performance in middle-aged and older adults: an ecological momentary cognitive assessment study. *JMIR mHealth uHealth.* (2020) 8:e19579. 10.2196/19579 32969829 PMC7545331

[15] Heine M van den Akker L Blikman L Hoekstra T Van Munster E Verschuren O Real-time assessment of fatigue in patients with multiple sclerosis: how does it relate to commonly used self-report fatigue questionnaires? *Arch Phys Med Rehabil.* (2016) 97:1887.e–94.e. 10.1016/j.apmr.2016.04.019 27233157

[16] Kratz A Murphy S Braley T. Pain, fatigue, and cognitive symptoms are temporally associated within but not across days in multiple sclerosis. *Arch Phys Med Rehabil.* (2017) 98:2151–9. 10.1016/j.apmr.2017.07.003 28729169 PMC5660935

[17] Goverover Y Kalmar J Gaudino-Goering E Shawaryn M Moore N Halper J The relation between subjective and objective measures of everyday life activities in persons with multiple sclerosis. *Arch Phys Med Rehabil.* (2005) 86:2303–8. 10.1016/j.apmr.2005.05.016 16344027

[18] Julian L Merluzzi N Mohr D. The relationship among depression, subjective cognitive impairment, and neuropsychological performance in multiple sclerosis. *Mult Scler J.* (2007) 13:81–6. 10.1177/1352458506070255 17294615

[19] Schmitt M Blum G. State/Trait Interactions. In: Zeigler-Hill V Shackelford TK editors. *Encyclopedia of personality and individual differences.* Cham: Springer (2020). p. 5206–9. 10.1007/978-3-319-24612-3_1922

[20] Harris P Taylor R Thielke R Payne J Gonzalez N Conde J. Research electronic data capture (REDCap)—a metadata-driven methodology and workflow process for providing translational research informatics support. *J Biomed Inform.* (2009) 42:377–81. 10.1016/j.jbi.2008.08.010 18929686 PMC2700030

[21] Harris P Taylor R Minor B Elliott V Fernandez M O’Neal L The REDCap consortium: building an international community of software platform partners. *J Biomed Infor.* (2019) 95:103208. 10.1016/j.jbi.2019.103208 31078660 PMC7254481

[22] Hensen B Mackworth-Young C Simwinga M Abdelmagid N Banda J Mavodza C Remote data collection for public health research in a COVID-19 era: ethical implications, challenges and opportunities. *Health Policy Plan.* (2021) 36:360–8. 10.1093/heapol/czaa158 33881138 PMC7928874

[23] Vesel C Rashidisabet H Zulueta J Stange J Duffecy J Hussain F Effects of mood and aging on keystroke dynamics metadata and their diurnal patterns in a large open-science sample: a BiAffect iOS study. *J Am Med Infor Assoc.* (2020) 27:1007–18. 10.1093/jamia/ocaa057 32467973 PMC7647317

[24] Zulueta J Piscitello A Rasic M Easter R Babu P Langenecker S Predicting mood disturbance severity with mobile phone keystroke metadata: a biaffect digital phenotyping study. *J Med Internet Res.* (2018) 20:e241. 10.2196/jmir.9775 30030209 PMC6076371

[25] Ross M Demos A Zulueta J Piscitello A Langenecker S McInnis M Naturalistic smartphone keyboard typing reflects processing speed and executive function. *Brain Behav.* (2021) 11:e2363. 10.1002/brb3.2363 34612605 PMC8613429

[26] Lechner-Scott J Kappos L Hofman M Polman C Ronner H Montalban X Can the expanded disability status scale be assessed by telephone? *Mult Scler J.* (2003) 9:154–9. 10.1191/1352458503ms884oa 12708811

[27] Smith A. *Symbol digit modalities test (SDMT).* Los Angeles, CA: Western Psychological Services (1982).

[28] Strober L DeLuca J Benedict R Jacobs A Cohen J Chiaravalloti N Symbol digit modalities test: a valid clinical trial endpoint for measuring cognition in multiple sclerosis. *Mult Scler J.* (2019) 25:1781–90. 10.1177/1352458518808204 30334474 PMC6826875

[29] Strober L Bruce J Arnett P Alschuler K Lebkuecher A Di Benedetto M A new look at an old test: normative data of the symbol digit modalities test–oral version. *Mult Scler Relat Disord.* (2020) 43:102154. 10.1016/j.msard.2020.102154 32450507

[30] Bot B Suver C Neto E Kellen M Klein A Bare C The mPower study, Parkinson disease mobile data collected using ResearchKit. *Sci Data.* (2016) 3:160011. 10.1038/sdata.2016.11 26938265 PMC4776701

[31] Carlsson A. Assessment of chronic pain. I. Aspects of the reliability and validity of the visual analogue scale. *Pain.* (1983) 16:87–101. 10.1016/0304-3959(83)90088-X 6602967

[32] Kleiman E. Understanding and analyzing multilevel data from real-time monitoring studies: an easily-accessible tutorial using R. *PsyArXiv.* [Preprint]. (2017). 10.31234/osf.io/xf2pw

[33] Bates D Mächler M Bolker B Walker S. Fitting linear mixed-effects models using lme4. *arXiv.* [preprint] arXiv:14065823. (2014). 10.18637/jss.v067.i01

[34] Kuznetsova A Brockhoff P Christensen R. lmerTest package: tests in linear mixed effects models. *J Stat Softw.* (2017) 82:1–26. 10.18637/jss.v082.i13

[35] Enders C Tofighi D. Centering predictor variables in cross-sectional multilevel models: a new look at an old issue. *Psychol Methods.* (2007) 12:121. 10.1037/1082-989X.12.2.121 17563168

[36] Feinstein A Magalhaes S Richard J Audet B Moore C. The link between multiple sclerosis and depression. *Nat Rev Neurol.* (2014) 10:507. 10.1038/nrneurol.2014.139 25112509

[37] Krupp L Elkins L. Fatigue and declines in cognitive functioning in multiple sclerosis. *Neurology.* (2000) 55:934–9. 10.1212/WNL.55.7.934 11061247

[38] Diamond B Johnson S Kaufman M Graves L. Relationships between information processing, depression, fatigue and cognition in multiple sclerosis. *Arch Clin Neuropsychol.* (2008) 23:189–99. 10.1016/j.acn.2007.10.002 18053682

[39] Leavitt V Brandstadter R Fabian M Katz Sand I Klineova S Krieger S Dissociable cognitive patterns related to depression and anxiety in multiple sclerosis. *Mult Scler J.* (2020) 26:1247–55. 10.1177/1352458519860319 31233379 PMC6928451

[40] Benson C Kerr B. Pain and cognition in multiple sclerosis. In: Taylor BK Finn DP editors. *Behavioral neurobiology of chronic pain.* Berlin: Springer (2014). p. 201–15. 10.1007/7854_2014_309 24850077

[41] Moore R Campbell L Delgadillo J Paolillo E Sundermann E Holden J Smartphone-based measurement of executive function in older adults with and without HIV. *Arch Clin Neuropsychol.* (2020) 35:347–57. 10.1093/arclin/acz084 31942632 PMC7244889

[42] Weizenbaum E Fulford D Torous J Pinsky E Kolachalama V Cronin-Golomb A. Smartphone-based neuropsychological assessment in Parkinson’s disease: feasibility, validity, and contextually driven variability in cognition. *J Int Neuropsychol Soc.* (2022) 28:401–13. 10.1017/S1355617721000503 33998438 PMC10474573

[43] Zhan A Mohan S Tarolli C Schneider R Adams J Sharma S Using smartphones and machine learning to quantify Parkinson disease severity: the mobile Parkinson disease score. *JAMA Neurol.* (2018) 75:876–80. 10.1001/jamaneurol.2018.0809 29582075 PMC5885192

[44] Torous J Staples P Barnett I Sandoval L Keshavan M Onnela J. Characterizing the clinical relevance of digital phenotyping data quality with applications to a cohort with schizophrenia. *NPJ Digit Med.* (2018) 1:15. 10.1038/s41746-018-0022-8 31304300 PMC6550248

[45] Goverover Y Chiaravalloti N DeLuca J. The relationship between self-awareness of neurobehavioral symptoms, cognitive functioning, and emotional symptoms in multiple sclerosis. *Mult Scler J.* (2005) 11:203–12. 10.1191/1352458505ms1153oa 15794396

[46] Feinstein A. Mood disorders in multiple sclerosis and the effects on cognition. *J Neurol Sci.* (2006) 245:63–6. 10.1016/j.jns.2005.08.020 16643952

[47] Grech L Kiropoulos L Kirby K Butler E Paine M Hester R. The effect of executive function on stress, depression, anxiety, and quality of life in multiple sclerosis. *J Clin Exp Neuropsychol.* (2015) 37:549–62. 10.1080/13803395.2015.1037723 26009936

[48] Arnett P Higginson C Randolph J. Depression in multiple sclerosis: relationship to planning ability. *J Int Neuropsychol Soc.* (2001) 7:665–74. 10.1017/S1355617701766027 11575588

[49] Kim E Lovera J Schaben L Melara J Bourdette D Whitham R. Novel method for measurement of fatigue in multiple sclerosis: real-time digital fatigue score. *J Rehabil Res Dev.* (2010) 47:477–84. 10.1682/JRRD.2009.09.0151 20803391

[50] Mobile Toolbox. *Mobile toolbox.* (2022). Available online at: https://mobiletoolbox.org/ (accessed August 30, 2022).

[51] Pew Resarch Center. *Mobile fact sheet.* (2021). Available online at: https://www.pewresearch.org/internet/fact-sheet/mobile/ (accessed August 30, 2022).

