# Supplementary material for: Real-time associations among MS symptoms and cognitive dysfunction using ecological momentary assessment
Source: Front Med (Lausanne). 2023 Jan 12;9:1049686. doi: 10.3389/fmed.2022.1049686 (PMC9877417; doi:10.3389/fmed.2022.1049686)
Supplement: Supplementary file 1 [file Table_1.DOCX]

Supplemental Table 1. Full model estimates for real-time associations between non-cognitive symptom ratings and mTMT-B performance.

|  | Standardized coefficient | 95% Confidence Intervals | *P* value |
| --- | --- | --- | --- |
| ***Fatigue*** | | | |
| (Intercept) | 0.02 | -0.14 to 0.17 | <0.001 |
| State fatigue | 0.03 | 0 to 0.05 | 0.183 |
| Trait fatigue | -0.05 | -0.21 to 0.12 | 0.581 |
| Measurement number | -0.26 | -0.3 to -0.23 | <0.001 |
| Age | 0.37 | 0.2 to 0.54 | <0.001 |
| State bilateral finger tapping performance (mean number of taps) | -0.01 | -0.04 to 0.01 | 0.331 |
| Trait bilateral finger tapping performance (mean number of taps) | -0.08 | -0.23 to 0.08 | 0.344 |
| State self-reported upper extremity weakness | 0.01 | -0.02 to 0.04 | 0.540 |
| Trait self-reported upper extremity weakness | 0.38 | 0.2 to 0.56 | <0.001 |
| State fatigue × trait fatigue | -0.01 | -0.04 to 0.02 | 0.522 |
| ***Depression*** | | | |
| (Intercept) | 0.01 | -0.14 to 0.17 | <0.001 |
| State depression | 0.08 | 0.04 to 0.12 | <0.001 |
| Trait depression | -0.04 | -0.19 to 0.11 | 0.605 |
| Measurement number | -0.26 | -0.29 to -0.23 | <0.001 |
| Age | 0.36 | 0.18 to 0.54 | <0.001 |
| State bilateral finger tapping performance (mean number of taps) | -0.01 | -0.04 to 0.02 | 0.444 |
| Trait bilateral finger tapping performance (mean number of taps) | -0.09 | -0.26 to 0.08 | 0.291 |
| State self-reported upper extremity weakness | 0.01 | -0.01 to 0.04 | 0.371 |
| Trait self-reported upper extremity weakness | 0.38 | 0.19 to 0.56 | <0.001 |
| State depression × trait depression | -0.04 | -0.06 to -0.01 | 0.005 |
| ***Anxiety*** | | | |
| (Intercept) | 0.02 | -0.13 to 0.17 | <0.001 |
| State anxiety | -2.44 × 10^-03^ | -0.04 to 0.03 | 0.776 |
| Trait anxiety | -0.13 | -0.3 to 0.05 | 0.160 |
| Measurement number | -0.26 | -0.29 to -0.23 | <0.001 |
| Age | 0.31 | 0.13 to 0.5 | 0.002 |
| State bilateral finger tapping performance (mean number of taps) | -0.01 | -0.04 to 0.01 | 0.330 |
| Trait bilateral finger tapping performance (mean number of taps) | -0.11 | -0.27 to 0.05 | 0.180 |
| State self-reported upper extremity weakness | 0.02 | -0.01 to 0.05 | 0.166 |
| Trait self-reported upper extremity weakness | 0.44 | 0.24 to 0.64 | <0.001 |
| State anxiety × trait anxiety | -0.01 | -0.04 to 0.02 | 0.401 |
| ***Pain*** | | | |
| (Intercept) | 0.01 | -0.14 to 0.17 | <0.001 |
| State pain | -0.01 | -0.04 to 0.02 | 0.096 |
| Trait pain | -0.09 | -0.29 to 0.1 | 0.349 |
| Measurement number | -0.26 | -0.3 to -0.23 | <0.001 |
| Age | 0.38 | 0.22 to 0.55 | <0.001 |
| State bilateral finger tapping performance (mean number of taps) | -0.01 | -0.04 to 0.02 | 0.368 |
| Trait bilateral finger tapping performance (mean number of taps) | -0.06 | -0.22 to 0.1 | 0.458 |
| State self-reported upper extremity weakness | 0.01 | -0.01 to 0.04 | 0.316 |
| Trait self-reported upper extremity weakness | 0.40 | 0.21 to 0.59 | <0.001 |
| State pain × trait pain | 0.04 | 0.005 to 0.07 | 0.024 |
| ***Number of hours slept in prior night*** | | | |
| (Intercept) | 0.005 | -0.14 to 0.15 | <0.001 |
| State hours slept | -0.02 | -0.05 to 0 | 0.899 |
| Trait hours slept | -0.12 | -0.26 to 0.02 | 0.104 |
| Measurement number | -0.26 | -0.3 to -0.23 | <0.001 |
| Age | 0.34 | 0.19 to 0.5 | <0.001 |
| State bilateral finger tapping performance (mean number of taps) | -0.01 | -0.04 to 0.02 | 0.347 |
| Trait bilateral finger tapping performance (mean number of taps) | -0.10 | -0.25 to 0.05 | 0.207 |
| State self-reported upper extremity weakness | 0.02 | -0.01 to 0.05 | 0.174 |
| Trait self-reported upper extremity weakness | 0.36 | 0.2 to 0.51 | <0.001 |
| State hours slept × trait hours slept | -0.002 | -0.03 to 0.02 | 0.883 |
| ***Difficulties falling asleep*** | | | |
| (Intercept) | -0.02 | -0.18 to 0.14 | <0.001 |
| Difficulty falling asleep (yes vs. no) | 0.08 | 0.002 to 0.16 | 0.045 |
| Measurement number | -0.27 | -0.3 to -0.24 | <0.001 |
| Age | 0.36 | 0.2 to 0.53 | <0.001 |
| State bilateral finger tapping performance (mean number of taps) | -0.01 | -0.04 to 0.02 | 0.347 |
| Trait bilateral finger tapping performance (mean number of taps) | -0.08 | -0.24 to 0.08 | 0.315 |
| State self-reported upper extremity weakness | 0.02 | -0.01 to 0.04 | 0.254 |
| Trait self-reported upper extremity weakness | 0.36 | 0.20 to 0.51 | <0.001 |

Note. All multilevel models included individual subject and day number as random intercepts, except for sleep variables which only included the subject’s intercept. Models were fit using the restricted maximum likelihood approach, which is the recommended default method by the R packages lme4 and lmerTest.

Supplemental Table 2. Full model estimates for real-time associations between non-cognitive symptom ratings and self-reported cognitive dysfunction.

|  | Standardized coefficient | 95% Confidence Intervals | *P* value |
| --- | --- | --- | --- |
| ***Fatigue*** | | | |
| (Intercept) | -0.03 | -0.25 to 0.18 | 0.006 |
| State fatigue | 0.17 | 0.15 to 0.20 | <0.001 |
| Trait fatigue | 0.22 | -0.01 to 0.45 | 0.066 |
| State depression | 0.07 | 0.04 to 0.10 | <0.001 |
| Trait depression | 0.15 | -0.04 to 0.34 | 0.137 |
| State fatigue × trait fatigue | -0.02 | -0.05 to 0.01 | 0.161 |
| ***Depression*** | | | |
| (Intercept) | -0.03 | -0.25 to 0.19 | <0.001 |
| State depression | 0.12 | 0.08 to 0.16 | <0.001 |
| Trait depression | 0.25 | 0.08 to 0.42 | 0.005 |
| State depression × trait depression | -0.01 | -0.04 to 0.01 | 0.273 |
| ***Anxiety*** | | | |
| (Intercept) | -0.02 | -0.24 to 0.20 | <0.001 |
| State anxiety | 0.08 | 0.05 to 0.11 | <0.001 |
| Trait anxiety | -0.10 | -0.39 to 0.19 | 0.493 |
| State depression | 0.09 | 0.06 to 0.12 | <0.001 |
| Trait depression | 0.32 | 0.06 to 0.58 | 0.021 |
| State anxiety × trait anxiety | -0.05 | -0.08 to -0.03 | <0.001 |
| ***Pain*** | | | |
| (Intercept) | -0.03 | -0.25 to 0.19 | <0.001 |
| State pain | 0.07 | 0.04 to 0.10 | <0.001 |
| Trait pain | 0.03 | -0.20 to 0.26 | 0.806 |
| State depression | 0.10 | 0.07 to 0.13 | <0.001 |
| Trait depression | 0.24 | 0.07 to 0.42 | 0.009 |
| State pain × trait pain | -0.02 | -0.05 to 0.01 | 0.223 |
| ***Number of hours slept in prior night*** | | | |
| (Intercept) | -0.02 | -0.24 to 0.19 | 0.043 |
| State hours slept | 0.01 | -0.02 to 0.04 | 0.253 |
| Trait hours slept | -0.10 | -0.30 to 0.11 | 0.361 |
| State depression | 0.12 | 0.09 to 0.15 | <0.001 |
| Trait depression | 0.22 | 0.05 to 0.38 | 0.012 |
| State hours slept × trait hours slept | -0.01 | -0.04 to 0.01 | 0.275 |
| ***Difficulties falling asleep*** | | | |
| (Intercept) | -0.02 | -0.24 to 0.20 | <0.001 |
| Difficulty falling asleep (yes vs. no) | -0.02 | -0.10 to 0.06 | 0.620 |
| State depression | 0.12 | 0.09 to 0.15 | <0.001 |
| Trait depression | 0.23 | 0.06 to 0.39 | 0.008 |

Note. All multilevel models included individual subject and day number as random intercepts, except for sleep variables which only included the subject’s intercept. Models were fit using the restricted maximum likelihood approach, which is the recommended default method by the R packages lme4 and lmerTest.
